# Bleb expansion requires transient membrane invaginations that sequester curvature-preferring proteins

**DOI:** 10.1073/pnas.2534871123

**Published:** 2026-05-20

**Authors:** Yuki Maekawa, Saori R. Yoshii, Noboru Mizushima, Junichi Ikenouchi

**Affiliations:** ^a^ Department of Biochemistry, Kyushu University Graduate School of Medical Sciences, Fukuoka 812-8582, Japan; ^b^ Department of Biochemistry and Molecular Biology, Graduate School of Medicine, The University of Tokyo, Tokyo 113-0033, Japan; ^c^ International Research Center for Neurointelligence, The University of Tokyo Institute for Advanced Study, The University of Tokyo, Tokyo 113-0033, Japan

**Keywords:** bleb, membrane curvature, plasma membrane invagination, curvature-preferring proteins, Caveolin-1

## Abstract

Bleb-based cell migration involves rapid plasma membrane expansion driven by intracellular pressure. How the membrane reorganizes its curvature and protein composition during this process remains unclear. Here, we identify a distinct inward membrane structure, termed the sub-bleb invagination (SBI), that forms de novo at the bleb base during expansion. SBIs display strong positive curvature and transiently sequester curvature-preferring integral membrane proteins such as Caveolin-1 and Piezo1 without endocytosis. Live-cell imaging shows that these proteins transiently accumulate at the SBIs in concert with bleb growth, indicating that bleb expansion dynamically redistributes membrane curvature and protein localization. Overexpression of curvature-preferring proteins markedly inhibited bleb enlargement and induced the aberrant formation of SBI-like membrane invaginations, suggesting that their excessive accumulation limits the membrane from unfurling. Our findings reveal a curvature-based mechanism for membrane protein sorting during bleb expansion and highlight how the interplay between membrane curvature and integral membrane protein organization shapes PM dynamics.

## Significance

Cells that migrate using blebs must rapidly expand their plasma membrane, yet how they reorganize membrane curvature and proteins during this process has remained unclear. Here, we identify a previously unrecognized membrane structure, the sub-bleb invagination (SBI), which forms at the bleb base during expansion. Using correlative light and electron microscopy, we show that SBIs are membrane invaginations with pronounced positive curvature that selectively sequester curvature-preferring membrane proteins such as Caveolin-1 and Piezo1, preventing their accumulation on the highly convex bleb surface. Notably, overexpression of Caveolin-1 suppresses bleb enlargement and impairs bleb-based amoeboid migration. These findings reveal a curvature-based mechanism that couples membrane remodeling with protein sorting, providing insight into how cells maintain mechanical flexibility during rapid shape changes.

The plasma membrane (PM) defines the boundary between the cytoplasm and the external environment, and its morphology is maintained by the underlying actin cortex. When the PM locally detaches from the cortex, cytoplasmic pressure drives its outward expansion, forming hemispherical blebs. Traditionally, blebs were viewed as passive protrusions that occur during processes accompanied by increased intracellular pressure, such as apoptosis and cytokinesis. However, recent studies have revealed that various cell types, including Dictyostelium amoebae, leukocytes, primordial germ cells (PGCs), and invasive cancer cells, actively use blebs for migration, a mode referred to as bleb-driven or bleb-based migration (1–3).

During bleb expansion, the PM must increase its surface area within seconds, despite being able to stretch by only 2 to 3% without rupturing (4). Recent work has demonstrated that preexisting tubular PM invaginations can unfold to provide this additional membrane, thereby enabling rapid bleb growth (5, 6). In zebrafish PGCs, Cdc42-dependent PM invaginations unfold at the cell front during migration, and disruption of this process severely impairs bleb formation and directional movement (7). These studies established that PM invaginations function as dynamic membrane reservoirs that support bleb expansion.

Curved membrane proteins play key roles in maintaining and unfurling PM invaginations. Cytosolic peripheral membrane proteins containing N-BAR or F-BAR domains, including amphiphysin and FBP17, can both sense and generate positive curvature, segregating membrane area into tubular invaginations that act as tension buffers (8, 9). Integral membrane proteins, such as Caveolin-1, also contribute to tension regulation via caveolae—Ω-shaped invaginations that flatten under mechanical stress to release stored membrane (10). However, while caveolins are physically constrained within the PM and BAR-domain proteins are highly dynamic, it remains unclear how these curvature-preferring proteins behave when the PM rapidly expands during bleb formation.

To address this question, we investigated how curvature-sensitive membrane proteins reorganize during bleb expansion. We found that, rather than simply relying on preexisting invaginations, cells form new inward PM invaginations at the bleb base during expansion. We term these structures sub-bleb invaginations (SBIs). SBIs are membrane regions characterized by high positive curvature that arise de novo and are enriched in membrane proteins with preference for positive curvature, Caveolin-1 and Piezo1 among them. Overexpression of these proteins suppresses bleb expansion, indicating that SBIs act as a membrane compartment that buffers local tension and curvature stress by temporarily isolating proteins with preference for positive curvature away from the expanding membrane. This finding suggests a reciprocal relationship between bleb growth and invagination formation. Previous studies showed that the unfolding of invaginations supplies membrane for bleb expansion, whereas our data indicate that bleb expansion can also induce inward invaginations. This dynamic redistribution of membrane curvature provides a perspective on how cells maintain mechanical homeostasis of the PM during rapid shape changes.

## Results

### SBIs form Concomitantly with Bleb Expansion.

**Fig. 1.**
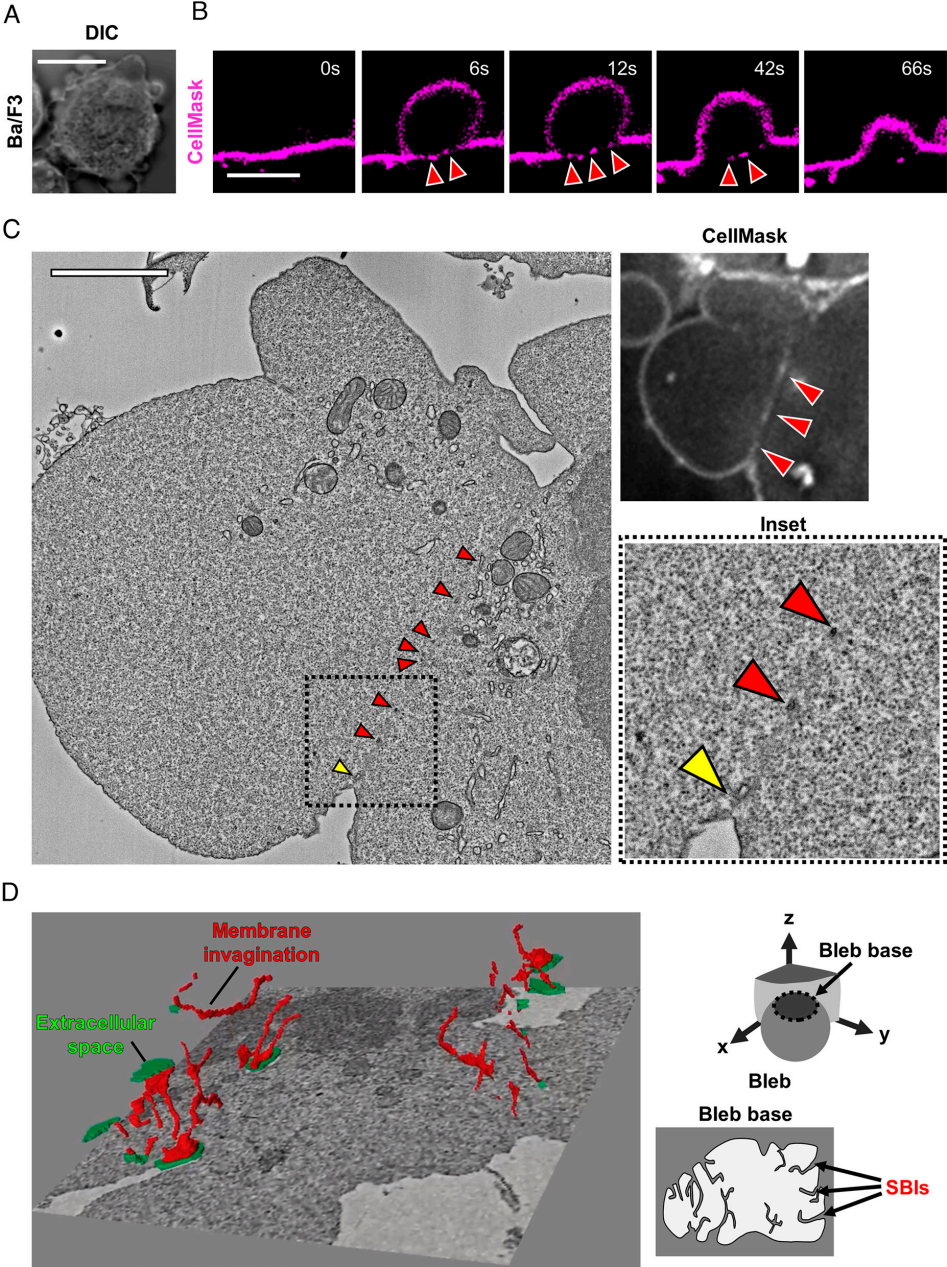
SBIs form concomitantly with bleb expansion. (*A*) Representative image of blebbing Ba/F3 cells stably expressing FLAG-tagged phospholipase D1 (PLD1). (Scale bar, 10 µm.) (*B*) Time-lapse images of a bleb in Ba/F3 cells expressing FLAG-PLD1 stained with CellMask. (Scale bar, 5 µm.) (*C*) Correlative light and electron microscopy (CLEM) images of a bleb in Ba/F3 cells expressing FLAG-PLD1 and stained with CellMask. Insets show higher-magnification views of the boxed region. Arrowheads indicate membrane structures corresponding to the CellMask signal at the bleb base. In the *Inset*, the yellow arrowhead indicates the base of the invagination where it remains connected to the PM, and the red arrowhead indicates a cross-section of the invaginated membrane structure. (Scale bar, 2 µm.) (*D*) Three-dimensional CLEM reconstruction of membrane structures at the bleb base (*Left*). The extracellular space is shown in green, and membrane structures at the bleb base are highlighted in red (Movie S2). A corresponding schematic illustration of SBIs is shown on the *Right*.

To examine PM dynamics during bleb formation, we analyzed Ba/F3 cells stained with CellMask to visualize the PM. Ba/F3 cells actively formed blebs upon stimulation with phorbol 12-myristate 13-acetate (PMA) (Fig. 1*A*). Time-lapse imaging revealed intermittent fluorescence along the boundary between the cytoplasmic compartment and the bleb, presumably the bleb base. These signals appeared concomitantly with the onset of bleb expansion and gradually disappeared during bleb retraction (Fig. 1*B* and Movie S1). Similar PM-derived signals at the bleb base were consistently observed in blebs formed by multiple cell lines, suggesting that this phenomenon represents a common feature of bleb dynamics (*SI Appendix*, Fig. S1*A* and Movie S1).

To exclude the possibility that this observation was specific to CellMask staining, we visualized the PM using several independent probes, including externally applied FITC-conjugated wheat germ agglutinin (FITC-WGA) and DiI C12:0, which label the outer leaflet, as well as the expressed PIP_2_-binding probe PLCδPH, which detects PIP_2_ on the inner leaflet. In all cases, similar discontinuous signals were detected at the bleb base (*SI Appendix*, Fig. S1 *B*–*D*), indicating that these structures are not staining artifacts.

To determine the ultrastructural basis of these signals, we performed CLEM. Electron microscopy revealed membrane invaginations at the bleb base that spatially coincided with the CellMask signal in Ba/F3 cells (Fig. 1*C*). Three-dimensional reconstruction of serial electron micrographs demonstrated that these structures were continuous with the PM at the bleb base and formed multiple narrow tubular invaginations extending inward from the cell surface (Fig. 1*D* and Movie S2).

The Ba/F3 cell line, which was used for CLEM analysis, stably expressed phospholipase D1 (PLD1) for the purpose of another study, but CellMask-based quantification showed no significant difference in the frequency of SBI-positive blebs between parental Ba/F3 cells and Ba/F3-PLD1 cells (*SI Appendix*, Fig. S1 *A* and *E*), indicating that the bleb-base membrane invaginations identified by CLEM are unlikely to be attributable to PLD1 expression.

Similar membrane invaginations continuous with the PM were also observed at the bleb base of HT1080 cells using high-speed three-dimensional imaging by spinning-disk confocal microscopy (*SI Appendix*, Fig. S1*F*).

Based on these observations, we designate these tubular PM invaginations formed at the bleb base during bleb expansion as SBIs. Notably, SBIs occasionally fragmented and were internalized into the cytoplasm, suggesting that their disappearance may be partly mediated by membrane internalization (*SI Appendix*, Fig. S1 *G* and *H*).

### FBP17 Localizes to SBIs.

To further characterize the dynamics of SBIs, we sought proteins that preferentially localize to these structures. BAR-domain proteins form curved dimers that bind membranes via their concave surfaces, enabling them to sense or generate membrane curvature. Because SBIs are expected to exhibit pronounced membrane curvature, we reasoned that specific BAR-domain proteins might selectively accumulate at these sites. We therefore screened a panel of fluorescently tagged BAR-domain proteins expressed in HT1080 cells for enrichment at the bleb base (*SI Appendix*, Fig. S2*A*).

**Fig. 2.**
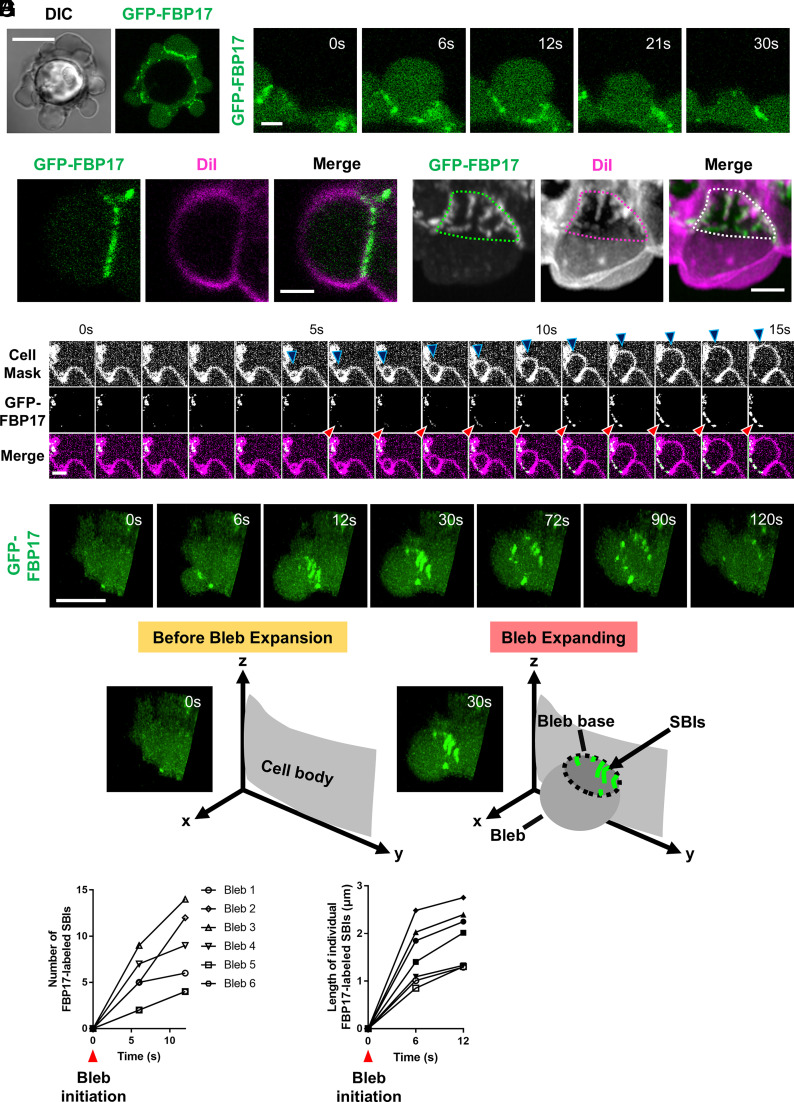
FBP17 localizes to SBIs. (*A*) Images of HT1080 cells expressing GFP-FBP17 forming blebs (*Left*; Scale bar, 10 µm) and time-lapse images of a bleb in HT1080 cells expressing GFP-FBP17 (*Right*; Scale bar, 2 µm). (*B*) Images of a DiI-stained bleb in HT1080 cells expressing GFP-FBP17. (Scale bar, 2 µm.) (*C*) High-speed time-lapse imaging of an expanding bleb in HT1080 cells expressing GFP-FBP17 and stained with CellMask. Red arrowheads indicate FBP17 accumulation, and blue arrowheads indicate the expanding bleb surface. (Scale bar, 2 µm.) (*D*) Three-dimensional reconstruction of a DiI-stained HT1080 cell expressing GFP-FBP17 showing a bleb protruding toward the viewer. The dotted line indicates the position of the bleb base. (Scale bar, 2 µm.) (*E*) Three-dimensional time-lapse images of a bleb in HT1080 cells expressing GFP-FBP17. (Scale bar, 5 µm.) (*F*) Quantification of the number of FBP17-labeled SBIs obtained from three-dimensional reconstructions, plotted at 6-s intervals (n = 6 blebs from 6 cells). (*G*) Quantification of temporal changes in the length of individual FBP17-labeled SBIs obtained from three-dimensional reconstructions, plotted at 6-s intervals (n = 7 structures from 4 cells).

Among the proteins examined, the F-BAR proteins FBP17 and Pstpip2 exhibited a distinctive localization pattern: both transiently accumulated at the bleb base coincident with the onset of bleb expansion and gradually dispersed over the course of bleb retraction (Fig. 2*A* and *SI Appendix*, Fig. S2*A* and Movie S3). To determine whether this accumulation corresponded to SBIs, the PM was visualized in GFP–FBP17–expressing cells using DiI C12:0 or CellMask. FBP17 signals overlapped precisely with SBIs (Fig. 2 *B*–*D*). High-speed imaging further revealed that FBP17 recruitment occurred concomitantly with the emergence of SBIs (Fig. 2*C* and Movie S4), and three-dimensional reconstruction confirmed that FBP17 specifically decorated individual SBI tubules (Fig. 2*D*).

Pstpip2 displayed nearly identical dynamics when coexpressed with FBP17 (*SI Appendix*, Fig. S2*B*). In contrast, other F-BAR proteins closely related to FBP17, including the TOCA family members CIP4 and TOCA1, as well as the F-BAR protein FCHo2 and the N-BAR protein Amphiphysin (AMPH), showed little or no enrichment at the SBIs (*SI Appendix*, Fig. S2*C*). Because only a subset of F-BAR proteins localized to SBIs, we next examined the structural determinants required for this targeting using truncated mutants of FBP17 (*SI Appendix*, Fig. S2*D*). Deletion of the F-BAR domain abolished localization to SBIs, demonstrating that this domain is essential for SBI association (*SI Appendix*, Fig. S2*E*). Notably, although full-length CIP4 failed to localize to SBIs, a truncated construct containing only the F-BAR domain (CIP4-F-BAR) was robustly recruited to these structures (*SI Appendix*, Fig. S2 *F* and *G*). These findings indicate that while the BAR domain provides the curvature-sensing module required for SBI targeting, regions outside the BAR domain modulate this localization, likely through interactions with other proteins or membrane components.

We next examined the N-BAR protein AMPH, which preferentially associates with membranes of higher curvature than those recognized by F-BAR domains. Although full-length AMPH did not accumulate at the SBIs, a truncated construct containing only the N-BAR domain (AMPH-N-BAR) transiently localized to the SBIs (*SI Appendix*, Fig. S2 *H* and *I*). However, unlike FBP17, AMPH-N-BAR had a shorter residence time at the SBIs and became enriched on the PM during bleb retraction (*SI Appendix*, Figs. S2 *J*–*L*), as previously reported (6). These differences likely reflect the distinct curvature preferences and membrane-binding properties of F-BAR and N-BAR domains (11–13).

To assess whether FBP17 can serve as a general marker of SBIs, GFP–FBP17 was expressed in multiple cell types and its localization was examined during bleb formation. In all cell lines analyzed in this study, FBP17 consistently accumulated at the bleb base, indicating that FBP17 functions as a broadly applicable marker of SBIs across diverse cellular contexts (*SI Appendix*, Fig. S3*A*). Furthermore, simultaneous imaging with markers of intracellular organelles revealed no detectable colocalization with FBP17-positive structures, further supporting the specificity of FBP17 as an SBI marker (*SI Appendix*, Fig. S3*B*).

Using FBP17 as a marker, we next performed three-dimensional live imaging to analyze SBI dynamics during bleb expansion (Fig. 2*E* and Movie S5). Quantitative analysis revealed that both the number and the length of SBIs increased progressively as the bleb expanded (Fig. 2 *F* and *G*). Previous studies have suggested that PM invaginations can function as membrane reservoirs that supply additional membrane surface during bleb expansion (5, 6). However, the behavior of SBIs differs fundamentally from that of such reservoir structures. Although membrane demand increases during bleb growth, SBIs form de novo at the bleb base and elongate inward as the bleb expands. This observation indicates that PM remodeling during bleb growth occurs in two opposing directions: outward protrusion of the bleb and simultaneous inward membrane invagination at the bleb base.

### Curvature-Preferring Membrane Proteins Accumulate at the SBIs.

**Fig. 3.**
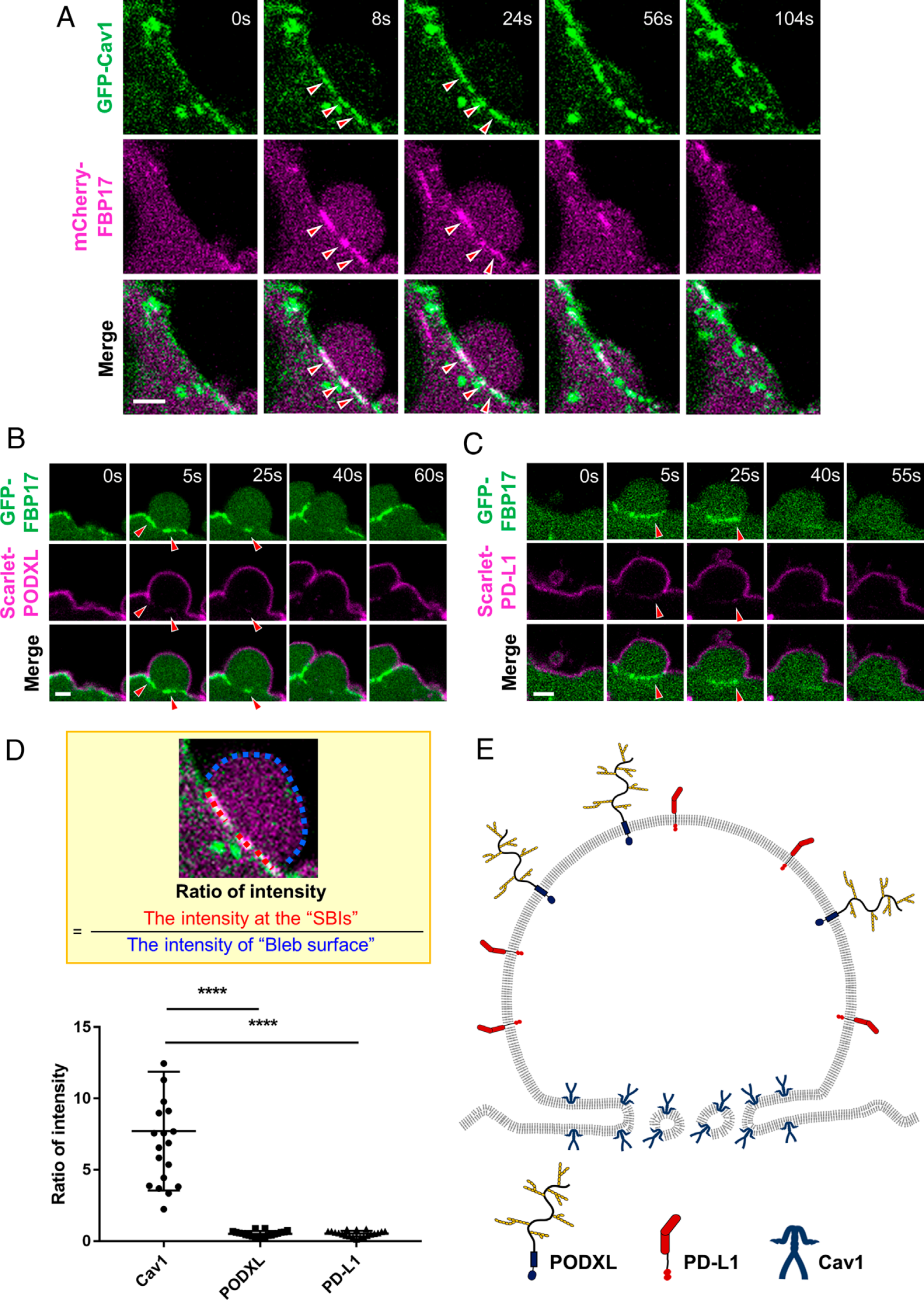
Cav1 is excluded from the PM of expanding blebs and accumulates in SBIs. (*A*) Time-lapse images of a bleb in HT1080 cells coexpressing GFP-Cav1 and mCherry-FBP17. Red arrowheads indicate Cav1 accumulation at the FBP17-labeled SBIs. (Scale bar, 2 µm.) (*B*) Time-lapse images of a bleb in HT1080 cells coexpressing GFP-FBP17 and Scarlet-PODXL. Red arrowheads indicate the FBP17-labeled SBIs. (Scale bar, 2 µm.) (*C*) Time-lapse images of a bleb in HT1080 cells coexpressing GFP-FBP17 and Scarlet-PD-L1. Red arrowheads indicate the FBP17-labeled SBIs. (Scale bar, 2 µm.) (*D*) Quantification of protein enrichment at the SBIs. As illustrated in the example shown within the yellow box, the mean fluorescence intensity of Cav1, PODXL, or PD-L1 at the FBP17-labeled SBIs was divided by that at the bleb surface to calculate the enrichment ratio. Data are shown as mean ± SD (n = 20 blebs per group). Statistical significance was assessed using one-way ANOVA followed by Dunnett’s multiple comparisons test. (*E*) Schematic of the localization of Cav1, PODXL, and PD-L1 in blebs. Because Cav1 is incompatible with the negative membrane curvature of the bleb protrusion, it is likely excluded from the bleb surface.

To characterize the properties of SBIs, we screened for membrane-associated proteins that localize to them. Among integral membrane proteins known to participate in PM invagination that function as membrane reservoirs, Caveolin-1 (Cav1) was markedly enriched at the bleb base, where it colocalized with FBP17-labeled SBIs, but excluded from the expanding bleb surface (Fig. 3 *A* and *D* and Movie S6). High-speed imaging showed that Cav1 accumulated at early SBIs before robust FBP17 enrichment became evident, although the two proteins subsequently colocalized (*SI Appendix*, Fig. S4*A* and Movie S7). A similar pattern of Cav1 accumulation was also observed in human melanoma M2 cells, which are well suited for observing blebs due to their active bleb formation (*SI Appendix*, Fig. S4*B*) (14).

In contrast, other membrane proteins such as PODXL (Podocalyxin-like protein 1), which typically localizes to microvillus-like membrane protrusions, and PD-L1 were distributed uniformly over the entire bleb surface without specific enrichment at the SBIs (Fig. 3 *B*–*D*), and their localization was clearly segregated from that of Cav1 (*SI Appendix*, Fig. S4 *C* and *D*). Cav1 is known to induce inward membrane curvature upon insertion into the PM and to preferentially associate with positively curved membrane regions (15, 16). Human melanoma M2 cells form numerous filopodia under serum-starved conditions and switch to blebbing upon serum stimulation (*SI Appendix*, Fig. S4*E*). When we compared Cav1 localization before and after bleb induction, we found that Cav1 was consistently excluded from filopodia and subsequently from the expanding bleb surface (*SI Appendix*, Fig. S4*F* and Movie S8). These observations indicate that blebs represent extreme outward membrane protrusions characterized by a curvature geometry that is unfavorable for Cav1. Therefore, the distinct distribution patterns of membrane proteins within blebs likely reflect their intrinsic curvature preferences. Membrane proteins that favor inward or positively curved membranes are physically mismatched with the highly convex surface of the bleb and are consequently excluded from it. As a result, SBIs may transiently serve as storage compartments that sequester curvature-preferring membrane proteins such as Cav1 (Fig. 3*E*).

### Dynamic Behavior and Curvature-Dependent Localization of Membrane Proteins Accumulated at the SBIs.

**Fig. 4.**
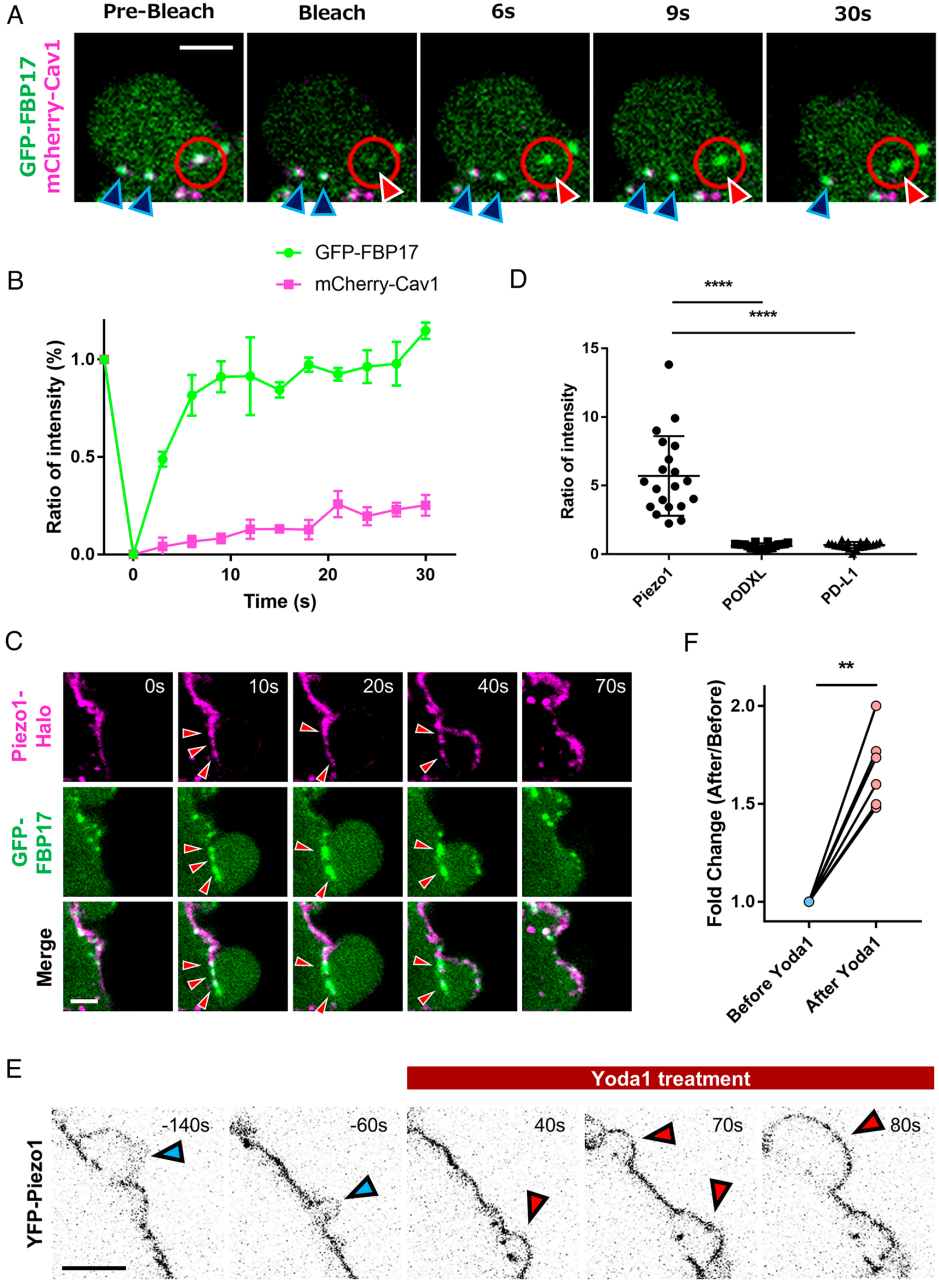
Membrane proteins accumulated in SBIs are unable to freely diffuse along the PM. (*A*) Representative FRAP time-lapse images of FBP17-labeled SBIs in HT1080 cells coexpressing GFP-FBP17 and mCherry-Cav1. Red circles mark the bleached area. Blue arrowheads indicate nonbleached SBIs, and red arrowheads indicate an SBI photobleached by laser irradiation. (Scale bar, 2 µm.) (*B*) Average FRAP recovery curves of GFP-FBP17 and mCherry-Cav1 at the SBIs in HT1080 cells (n = 3 independent experiments). (*C*) Time-lapse images of a bleb in HT1080 cells coexpressing GFP-FBP17 and Piezo1-Halo. Red arrowheads indicate Piezo1 accumulation at the FBP17-labeled SBIs. (Scale bar, 2 µm.) (*D*) Quantification of protein enrichment at the SBIs. As described in Fig. 3*D*, the mean fluorescence intensity of Piezo1, PODXL, or PD-L1 at the FBP17-labeled SBIs was divided by that at the bleb surface to calculate the enrichment ratio. Data are shown as mean ± SD (n = 20 blebs per group). Statistical significance was assessed using one-way ANOVA followed by Dunnett’s multiple comparisons test. (*E*) Time-lapse images of YFP–Piezo1 before and after treatment with 50 µM Yoda1 displayed as inverted projections. Blue arrowheads indicate blebs before Yoda1 treatment, and red arrowheads indicate blebs after Yoda1 treatment. (Scale bar, 5 µm.) (*F*) Quantification of Piezo1 fluorescence intensity at the bleb surface before and after Yoda1 treatment. For each cell, fluorescence intensity was measured on ≥3 blebs before and ≥3 blebs after treatment, and the mean values were calculated for each condition. The mean intensity before treatment was normalized to 1 for each cell, and the mean intensity after treatment was expressed as a fold change. Data are shown as mean ± SD (n = 7 cells). Statistical significance was assessed using a two-tailed paired Student’s *t* test. *P* = 0.0015.

To analyze the dynamics of proteins associated with SBIs, we performed fluorescence recovery after photobleaching (FRAP) experiments for FBP17 and Cav1. The cytosolic protein FBP17 exhibited rapid fluorescence recovery, whereas the membrane protein Cav1 showed low mobility within SBIs and displayed almost no recovery (Fig. 4 *A* and *B* and Movie S9). These results indicate that FBP17 undergoes rapid turnover through dynamic molecular exchange, while Cav1 is stably retained at the SBIs, indirectly supporting the hypothesis that SBIs function as temporary storage compartments for specific membrane proteins.

Next, we examined another curvature-preferring membrane protein, Piezo1, which senses membrane curvature through its trimeric structure and is excluded from negatively curved structures such as filopodia (17, 18). During bleb formation, Piezo1 was excluded from the PM of the expanding protrusive region, while being clearly enriched at the FBP17-labeled SBIs, mirroring Cav1 localization (Fig. 4 *C* and *D* and Movie S10). Consistently, Piezo1 exhibited a localization pattern distinct from those of PODXL and PD-L1 (*SI Appendix*, Fig. S4 *G* and *H*), and a similar distribution was also observed in human melanoma M2 cells (*SI Appendix*, Fig. S4*I*). Notably, as with Cav1, Piezo1 was invariably excluded from filopodia and other protrusive membrane structures both before and after bleb formation (*SI Appendix*, Fig. S4*J* and Movie S11).

To test whether the localization of Piezo1 depends on its curvature preference, we treated Piezo1-YFP–expressing cells with Yoda1, a Piezo1 activator that stabilizes its open conformation and shifts its preference toward flat membrane regions (17). Before Yoda1 treatment, Piezo1 was enriched at the SBIs and largely excluded from the bleb surface (Fig. 4*E*, blue arrowheads). Upon Yoda1 addition, however, the amount of Piezo1 on the bleb surface increased significantly (Fig. 4*E*, red arrowheads; Fig. 4*F* and Movie S12). These results demonstrate that activation of Piezo1 reduces its preference for positive curvature, thereby increasing its distribution on the PM of the expanding bleb and reducing its relative enrichment at SBIs. Together, these findings indicate that the accumulation of membrane proteins such as Cav1 and Piezo1 at SBIs depends on their intrinsic curvature preference. The highly convex PM of the expanding bleb excludes curvature-preferring proteins, which are instead preferentially redistributed to SBIs.

### Overexpression of Curvature-Preferring Membrane Proteins Suppresses Bleb formation.

**Fig. 5.**
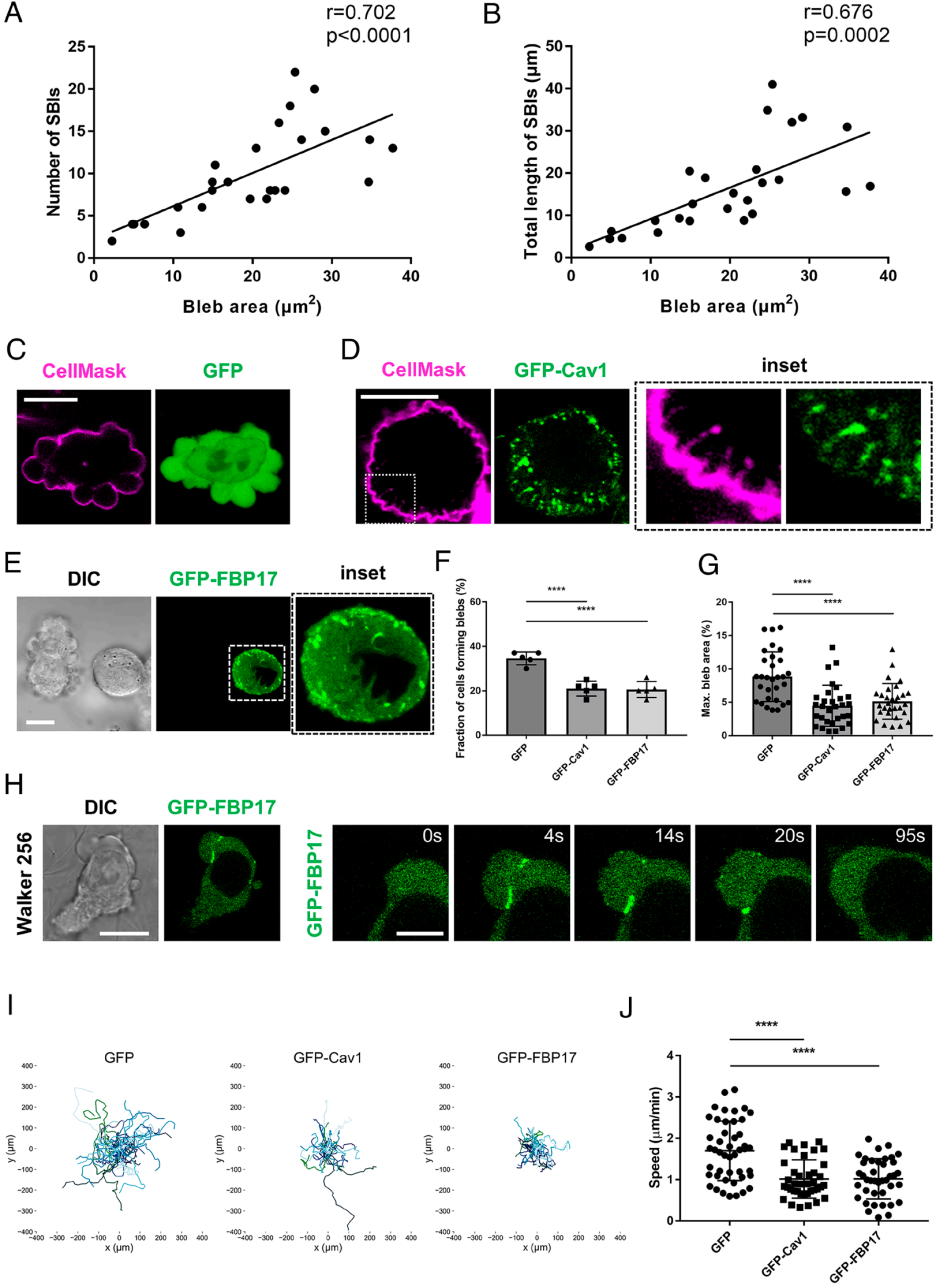
Overexpression of positive curvature-preferring membrane proteins limits bleb expansion. (*A*) Correlation between the number of FBP17-labeled SBIs and the bleb area in three-dimensional reconstructions of HT1080 cells expressing GFP-FBP17 (n = 26 blebs from 17 cells). (*B*) Correlation between the total length of FBP17-labeled SBIs and the bleb area in three-dimensional reconstructions of HT1080 cells expressing GFP-FBP17 (n = 26 blebs from 17 cells). (*C*) Images of HT1080 cells expressing GFP and stained with CellMask (1:1,000 dilution). (Scale bar, 10 µm.) (*D*) Images of HT1080 cells overexpressing GFP-Cav1 and stained with CellMask (1:1,000 dilution). Numerous tubular invaginations were induced on the PM (*Inset*). (Scale bar, 10 µm.) (*E*) Images of HT1080 cells overexpressing GFP-FBP17. Numerous tubular invaginations were induced on the PM (*Inset*). (Scale bar, 10 µm.) (*F*) The fraction of bleb-forming cells was quantified in HT1080 cells overexpressing GFP, GFP-Cav1, or GFP-FBP17 by counting 100 cells for each condition (n = 5 independent experiments). Statistical significance was assessed using one-way ANOVA followed by Dunnett’s multiple comparisons test. (*G*) Quantification of the maximum bleb area in HT1080 cells overexpressing GFP, GFP-Cav1, or GFP-FBP17. To normalize for differences in overall cell size, the bleb cross-sectional area was divided by the total cell cross-sectional area (n = 30 cells). Statistical significance was assessed using one-way ANOVA followed by Dunnett’s multiple comparisons test. (*H*) Images of Walker 256 cells expressing GFP-FBP17 (*Left*; Scale bar, 10 µm) and time-lapse images of a representative bleb (*Right*; Scale bar, 2 µm). (*I*) Tracking of cell migration in Walker 256 cells overexpressing GFP, GFP-Cav1, or GFP-FBP17. Individual cells were tracked for 3 h 30 min, and the trajectories of GFP-expressing (n = 46), GFP-Cav1–expressing (n = 39), and GFP-FBP17–expressing (n = 40) cells were plotted. (*J*) Mean migration speed was calculated by dividing the total distance traveled (µm) by the observation time (min) for each cell. Data are shown as mean ± SD (GFP, n = 46; GFP-Cav1, n = 39; GFP-FBP17, n = 40). Statistical significance was assessed using one-way ANOVA followed by Dunnett’s multiple comparisons test.

During bleb expansion, membrane proteins that prefer positive curvature, such as Cav1 and Piezo1, are excluded from the highly convex bleb surface and accumulate within SBIs. This suggests that the formation of large blebs requires efficient exclusion of curvature-preferring membrane proteins, accompanied by an increased number of SBIs that accommodate them. Indeed, quantitative analysis revealed a positive correlation between bleb size and both the number and total length of FBP17-labeled SBIs (Fig. 5 *A* and *B*). Based on these findings, we consider the PM as a two-dimensional fluid in which membrane proteins dissolve according to their curvature preference. Proteins with strong positive curvature, such as Cav1 and Piezo1, are energetically incompatible with the relatively flat or even negatively curved surface of expanding blebs. To minimize curvature mismatch, these proteins are excluded from the bleb surface and enriched in highly invaginated membrane domains such as SBIs, which provide local positive curvature that accommodates them. If the PM has a limited capacity to accommodate positive curvature membrane proteins, we reasoned that artificial overexpression of such proteins would increase their surface density beyond this “solubility limit,” necessitating the formation of numerous curvature-stabilized invaginations similar to SBIs. To test this hypothesis, we examined the phenotype of cells overexpressing Cav1. Interestingly, we found that overexpression of Cav1 induced excessive tubular invaginations in the PM (Fig. 5 *C* and *D*). A similar phenotype was observed upon FBP17 overexpression (Fig. 5*E*). In these cells, the frequency and average size of blebs were significantly reduced (Fig. 5 *F* and *G*). These results indicate that positive curvature–preferring membrane proteins inhibit bleb expansion by diverting the PM into invaginated structures, thereby limiting the membrane supply required for bleb growth. To assess the functional consequence of this mechanism in amoeboid migration, we next analyzed Walker 256 carcinoma cells, which undergo bleb-based amoeboid migration (19). FBP17-labeled SBIs were also observed during bleb expansion in Walker 256 cells (Fig. 5*H*). Remarkably, overexpression of Cav1 or FBP17 in these cells markedly suppressed amoeboid migration, leading to a substantial reduction in cell motility (Fig. 5 *I* and *J*).

Together, these findings indicate that the overabundance of curvature-preferring membrane proteins disrupts the balance between membrane curvature and availability required for efficient bleb expansion, thereby limiting the mechanical flexibility essential for amoeboid migration.

## Discussion

In this study, we identified a transient PM invagination that forms at the base of expanding blebs, which we term SBI. Unlike previously described inward PM invaginations proposed to serve as membrane reservoirs, SBIs are generated dynamically during bleb expansion. Membrane proteins that preferentially associate with positive curvature, including Cav1 and Piezo1, were excluded from the highly convex bleb surface and instead accumulated in SBIs.

Quantitative analysis revealed that both the number and length of SBIs positively correlate with bleb size. Importantly, this correlation does not indicate that SBIs are required for bleb initiation. Rather, SBIs appear to arise as transient membrane rearrangements during the rapid membrane deformation that accompanies bleb growth. In this context, we propose that SBIs serve as membrane compartments that temporarily sequester proteins incompatible with the highly convex bleb surface. By redistributing curvature-preferring proteins away from the bleb membrane, SBIs may help buffer membrane tension and thereby facilitate continued bleb expansion.

Consistent with this interpretation, excessive accumulation of curvature-preferring membrane proteins promoted extensive tubular invaginations of the PM and reduced both the frequency and size of blebs. These conditions were accompanied by impaired bleb-based amoeboid migration, suggesting that the quantitative balance of curvature-sensitive membrane proteins directly influences the membrane available for bleb expansion.

How can we reconcile these findings with the long-standing view that membrane invaginations function as membrane reservoirs? Although unfolding of preexisting invaginations may supply additional surface area during bleb expansion, our data indicate that membrane components are not incorporated uniformly into the expanding bleb membrane. Instead, proteins incompatible with the highly convex bleb membrane appear to be selectively redistributed to SBIs. Previous studies have suggested that bleb formation can be associated with filopodia-like protrusions (20, 21), raising the possibility that bleb expansion involves not only unfolding of membrane invaginations but also reorganization of preexisting protrusive structures. Together, these observations suggest that bleb growth is accompanied by dynamic sorting of membrane components according to their curvature preference, resulting in the formation of SBIs at the bleb base.

What mechanisms might drive SBI formation? Bleb expansion is powered by cytoplasmic influx that pushes the PM outward, generating elevated membrane tension across the bleb surface (22). However, the bleb base likely represents a mechanically distinct boundary between the highly stretched bleb membrane and the actin cortex–supported PM, where membrane curvature changes abruptly and the local tension field becomes heterogeneous. In this region, local accumulation of curvature-preferring membrane components may lower the energetic barrier for inward membrane bending, thereby permitting transient SBI formation even during bleb expansion. Consistent with this idea, Cav1 accumulated at the bleb base prior to FBP17 (*SI Appendix*, Fig. S4*A*), and clustering of curvature-preferring membrane proteins promoted membrane invagination.

Structural factors may further facilitate this process. During bleb expansion, the PM detaches from the cortical actin network, whereas portions of the preexisting actin cortex can remain at the original cell boundary. Our CLEM and fluorescence imaging analysis with Lifeact supports this view by revealing residual actin cortex in the bleb base region after membrane detachment (*SI Appendix*, Fig. S5 *A* and *B*). SBIs frequently appeared to extend into regions adjacent to this residual cortex, suggesting that membrane–cortex attachment may act as a nucleation point for membrane bending. In addition, high curvature at the bleb edge may promote local recruitment of FBP17, thereby facilitating SBI formation. Once recruited, FBP17 may further promote membrane deformation and elongation, contributing not only to SBI initiation but also to subsequent growth and stabilization.

In addition to these mechanical and structural factors, cytoplasmic heterogeneity generated during bleb expansion may provide an additional driving force for SBI formation. We previously reported that proteins such as Mena become enriched within expanding blebs and that the cytoplasmic composition differs substantially between the bleb and the cell body (23, 24). Notably, SBIs frequently form at the interface between these two cytoplasmic domains, suggesting that membrane deformation may occur preferentially at this biochemical boundary (*SI Appendix*, Fig. S5 *C* and *D*). Biochemical phase separation provides a plausible mechanism for such membrane remodeling. In model membrane systems such as giant unilamellar vesicles, liquid–liquid phase separation of internal polymers induces membrane tubulation at droplet interfaces through changes in wetting behavior and interfacial tension (25). Analogously, protein condensation within blebs could generate interfacial tension differences that promote membrane invagination at the bleb–cell body boundary. Consistent with this possibility, FBP17-labeled SBIs frequently formed adjacent to regions enriched in Mena. In addition, cytoplasmic Ca^2+^ levels rise markedly within expanding blebs (24). Elevated Ca^2+^ may further promote macromolecular condensation by bridging negatively charged lipids and proteins and reducing electrostatic repulsion among macromolecules, thereby generating capillary forces capable of pulling the membrane inward. The residual actin cortex beneath the bleb membrane may also help maintain this cytoplasmic heterogeneity by partially restricting exchange between the bleb and the cell body.

SBIs eventually disappear as blebs retract. We interpret SBI dissolution in the context of cortical reassembly during bleb termination. As bleb growth ceases and the actin cortex is reassembled beneath the membrane, the membrane–cortex attachment is restored and the local conditions that support SBI maintenance are lost. Under these conditions, curvature-preferring proteins redistribute along the PM, leading to dissolution of SBI structures. In some cases, SBIs also appeared to fragment and become internalized as vesicular structures (*SI Appendix*, Fig. S1 *F* and *G*), although the mechanism of this process remains unclear and may involve membrane remodeling and scission during bleb retraction.

Our findings further demonstrate that the quantitative balance of curvature-preferring membrane proteins strongly influences bleb expansion and cell migration. The ability of cells to regulate the abundance and spatial distribution of these proteins may therefore represent an important determinant of membrane deformability and bleb-based motility. Cancer cells adopt multiple migration modes, including bleb-based amoeboid migration and mesenchymal migration, and some cells switch from mesenchymal to bleb-based motility under hypoxic conditions (26, 27). Previous work has shown that increased cortical tension and reduced cell–matrix adhesion contribute to this transition (28). Our results suggest that the abundance of curvature-preferring membrane proteins may represent an additional factor influencing whether cells adopt bleb-based migration.

This mechanism may also contribute to cell polarity. In mesenchymal migration, Cav1 and Piezo1 preferentially accumulate at the cell rear (29–31). Enrichment of curvature-preferring proteins in the rear membrane may locally reduce membrane deformability, thereby limiting protrusion formation and reinforcing front–rear polarity.

Finally, SBIs may also function as curvature-dependent signaling platforms. Blebs have been implicated not only in amoeboid migration but also in cancer cell survival and chemoresistance (32, 33). For example, septins accumulating at the bleb neck can act as signaling hubs conferring anoikis resistance (34). By analogy, SBIs may serve as membrane compartments that locally concentrate receptors and signaling molecules in a curvature-dependent manner.

Taken together, our findings suggest that SBIs arise at the interface between mechanically and biochemically distinct cellular domains during bleb expansion, where membrane curvature, protein sorting, cytoskeletal organization, and cytoplasmic phase behavior converge to regulate PM remodeling. Understanding how these processes cooperate to generate SBIs will provide insights into how dynamic membrane remodeling supports cell migration, polarity, and pathological processes such as cancer invasion and drug resistance.

## Materials and Methods

### Cells and Reagents.

DLD1, HEK293, HeLa, 786-O, A498, 4T1, and A375 cells were obtained from the American Type Culture Collection (ATCC). Caki-1 and HT1080 cells were obtained from the Japanese Collection of Research Bioresources (JCRB) Cell Bank. Ba/F3 cells were obtained from the RIKEN Cell Bank (RCB4476). Walker 256 cells were kindly provided by Dr. Jolanta Sroka (Jagiellonian University, Poland), and M2 cells were kindly provided by Dr. Yasutaka Ohta (Kitasato University, Japan).

AccuDia DMEM 2 (Cat#05919) and AccuDia RPMI1640 2 (Cat#05918) were purchased from Shimadzu Diagnostics Corporation. Fetal bovine serum (FBS; Cat#S-001A-BR) was purchased from Life Science Production. 200 mM L-Glutamine Stock Solution (Cat#16948-04) and Agarose-LM (Cat#01161-54) were purchased from Nacalai Tesque. Opti-MEM (Cat#31985070), HBSS (Cat#14065056), DiI C12(3) (Cat#D383), CellMask Plasma Membrane Stain (Cat#C10046), LysoTracker Red DND-99 (Cat#L7528), and Wheat Germ Agglutinin (WGA) Alexa Fluor 488 (Cat#W11261) were purchased from Thermo Fisher Scientific. HaloTag TMR Ligand (Cat#G8252) was purchased from Promega. Phorbol 12-myristate 13-acetate (PMA; 168-23593) was obtained from Fujifilm Wako. Native Collagen Acidic Solution I-AC (Cat#IAC-50) and MitoBright LT Red (Cat#349-92073) were purchased from FUJIFILM Wako Chemicals. PEI MAX (Cat#24765-1) was purchased from Polysciences. CK666 (Cat#182515-25MG) and Yoda1 (Cat#SML1558-5MG) were purchased from Sigma-Aldrich. Recombinant Murine IL-3 (Cat#213-13) was purchased from Funakoshi. The following expression vectors were purchased from Addgene: FBP17-pmCherryC1 (Plasmid #27688), Cav1-GFP (Plasmid #14433), CAV1-mCherry (Plasmid #27705), PIEZO1-HaloTag (Plasmid #207834), mCh-Sec61β (Plasmid #49155), mCh-Rab5 (Plasmid #49201), mCh-Rab7A (Plasmid #61804), GFP-rab11 WT (Plasmid #12674), CIP4-pmCherryC1 (Plasmid #27685), FCHo2-pmCherryC1 (Plasmid #27686), pCAG-AMPH1-BAR-mCherry (Plasmid #85130), Amph1-pmCherryN1 (Plasmid #27692), BIN1-pmCherryN1 (Plasmid #27693), SNX9-pmCherryC1 (Plasmid #27678), and mCherry-TUBA (Plasmid #129622). cDNAs encoding full-length or truncated mutants of mouse FBP17 (ΔSH3, ΔF-BAR), and those encoding mouse CIP4-F-BAR, mouse PODXL, mouse Fer, mouse Pstpip2, mouse RICH2, mouse ACAP2, mouse APPL1, mouse ASAP1, mouse IRSp53, mouse PLCδPH, human PD-L1, human srGAP1, human Pacsin2, human RICH1, human EndophilinA2, human ARHGAP26, human MIM, and human Mena were amplified by RT-PCR, fused to the sequences encoding EGFP or mScarlet, and ligated into the pCAGGS-neo vector. The Scarlet-TOCA1 construct was purchased from Addgene (Plasmid #33030) and was cloned into the pCAGGS-neo vector. A FLAG-tagged human PLD1 cDNA was cloned from HeLa cells and inserted into pMRX-IPU vector (35). The expression vector for YFP-Piezo1 was a kind gift from Dr. David J. Beech (School of Medicine, University of Leeds, UK).

### Cell Culture.

DLD1, HEK293, HT1080, HeLa, 786-O, Caki-1, A498, A375, and M2 cells were cultured in DMEM supplemented with 10% (v/v) fetal bovine serum (FBS) at 37 °C in 5% CO2. 4T1 and Walker 256 cells were cultured in RPMI1640 supplemented with 10% (v/v) FBS under the same incubation conditions. Ba/F3 cells were cultured in RPMI1640 supplemented with 10% (v/v) FBS and 5 ng/mL IL-3. To maintain their suspension state, Ba/F3 and Walker 256 cells were cultured in ultra-low attachment flasks (Corning Life Sciences, Cat#3814) as previously described (36). All cell lines were routinely tested and confirmed to be negative for mycoplasma contamination.

### Gene Expression.

Gene transfection into adherent cells was performed using the polyethyleneimine (PEI) method. Opti-MEM medium equivalent to 5% of the total culture volume was mixed with plasmid DNA and PEI to final concentrations of 1 µg/mL and 3 µg/mL, respectively. The DNA-PEI mixture was incubated at room temperature for 15 min and then added to the cultured cells, which were subsequently incubated at 37 °C. For HT1080 cells, the culture medium was completely replaced 4 h after the addition of the DNA–PEI mixture, and incubation was continued at 37 °C.

Walker 256 and Ba/F3 cells were transfected by electroporation. A total of 1 × 10^6^ cells were suspended in 100 µL of Opti-MEM containing 10 µg of plasmid DNA and transferred into a 2 mm-gap cuvette (NEPAGENE, Cat#EC-002S). Electroporation was performed using a NEPA21-S electroporator (NEPAGENE) under the following conditions: Poring pulse (150 V, pulse length = 2.5 ms, pulse interval = 50 ms, number of pulses = 2, decay rate = 10%) and Transfer pulse (20 V, pulse length = 50 ms, pulse interval = 50 ms, number of pulses = 5, decay rate = 40%). Immediately after electroporation, cells were transferred into RPMI1640 supplemented with 10% FBS and incubated for 24 h at 37 °C in 5% CO_2_.

### Live Imaging.

DLD1, M2, and HEK293 cells were cultured on 35-mm glass-bottom dishes (Matsunami, Cat#D11130H) for at least 6 h at 37 °C in 5% CO_2_ before imaging. HeLa cells were imaged 2 h after seeding under the same conditions (37). HT1080 cells were prepared as previously described (21); briefly, cells were cultured on 35-mm glass-bottom dishes for 10 to 16 h, treated with 200 µM CK666 for at least 1 h, and then used for bleb imaging. A375, 786-O, A498, and Caki-1 cells were embedded in DMEM containing 2.5 mg/mL collagen and seeded onto glass-bottom dishes. After the collagen had completely polymerized, 1 mL of prewarmed DMEM (37 °C) was added before imaging. Ba/F3 cells were stimulated with 750 nM PMA for 10 min. A 5 µL aliquot of the cell suspension was placed onto a glass-bottom dish and overlaid with a thin layer of agarose gel prior to imaging.

Fluorescence live-cell imaging was performed using an LSM 900 confocal laser-scanning microscope (ZEISS) equipped with a 63× oil-immersion objective (NA 1.40) and a heating stage maintained at 37 °C. Three-dimensional fluorescence imaging (Fig. 2 *D* and *E* and *SI Appendix*, Fig. S1*F*) or high-speed imaging (Fig. 2*C* and *SI Appendix*, Figs. S2*L* and S4*A* and Movies S4 and S7) was conducted using a spinning-disk confocal microscope (Dragonfly 200; Oxford Instruments) mounted on an inverted microscope (IX83; Olympus) equipped with a 100× oil-immersion objective and a 37 °C heating stage. Three-dimensional reconstruction of the Dragonfly 200 datasets was performed using Imaris software (Bitplane), and fluorescence intensity analysis was carried out using ImageJ/Fiji.

Tracking of Walker 256 cells was performed under confinement using a 1% agarose pad. Bleb-based migration was imaged for 3.5 h with a 10× objective lens on a BZ-X810 microscope (KEYENCE). Glass-bottom dishes were coated with 1% BSA in PBS for 1 h and washed three times with PBS. Agarose-LM (0.2 g) was dissolved in 10 mL Milli-Q water, mixed with 10 mL 2 × HBSS, and 4 mL of the mixture was poured onto the coated dish. After gelation at room temperature, the dish was placed at 37 °C in 5% CO_2_’ overnight. Before imaging, small holes were made in the agarose pad using a pipette tip, and 100 µL of cell suspension was added into the holes. The pad was then gently lifted to allow the cells to move underneath. Excess medium was completely removed. Following confinement, cells were incubated for 30 min at 37 °C in 5% CO_2_ before imaging.

For serum-stimulation-induced bleb formation in M2 cells, cells were precultured in FBS-free DMEM for 10 to 16 h, and 20% FBS-DMEM (equal volume to the existing medium) was added during live imaging (*SI Appendix*, Fig. S4 *E*, *F* and *J* and Movies S8 and S11).

### Three-Dimensional CLEM.

Three-dimensional CLEM was performed as previously described (38). For CLEM analysis, 3 μL of stained cell suspension was placed onto a carbon-coated finder grid mounted on a glass-bottom dish (TCI-3922-035R; Iwaki). The sample was covered with a thin layer of 1% agarose prepared in serum-free DMEM. The area surrounding the thin agarose layer was then filled with liquid low-melting-point agarose pre-equilibrated at 37 °C. The agarose was allowed to solidify at room temperature, after which the dish was returned to a CO2 incubator for 20 min. Cells were fixed with 2% paraformaldehyde (26126-54, Nacalai Tesque) and 0.5% glutaraldehyde (G018/1, Nisshin EM). Following washes with 0.1 M phosphate buffer (pH 7.4, RM102-5L, LSI Medience), fluorescence images were acquired using a DeltaVision Elite imaging system equipped with a 60× oil-immersion objective lens prior to electron microscopy processing. After acquiring fluorescence images, cells were fixed again overnight with 2.5% glutaraldehyde in 0.1 M cacodylate buffer (pH 7.4; 37237-35, Nacalai Tesque). Cells were then postfixed with 1% osmium tetroxide (3020-4; Nisshin EM) in 0.065 M cacodylate buffer for 2 h at 4 °C. After treatment with 3% uranyl acetate for 1 h, the samples were dehydrated through 100% ethanol and embedded in Epon resin (EPON 812; Nisshin EM). The area containing the target cells was trimmed using a razor blade. Ultrathin serial sections (50 nm thickness) were prepared using a diamond knife with an ultrajumbo boat (Ultrajumbo 45°, Diatome) mounted on an ultramicrotome (UC7; Leica). Scanning electron microscopy (SEM) images were acquired using an electron microscope (JSM7900F; JEOL). The acquired SEM images were aligned in sequential order using Measurement Advisor 4.1.14.0 software (System In Frontier Inc.). Images were manually stacked using Stacker NEO TEMography.com 3.3.4.0 software (System In Frontier Inc.). Membrane structures were manually segmented by tracing the boundaries in each serial section using Image-Pro 10.0.7 software (Media Cybernetics).

### FRAP Analyses.

Fluorescence recovery after photobleaching (FRAP) experiments were performed using an LSM 900 confocal laser-scanning microscope (ZEISS) equipped with a 63× oil-immersion objective lens (NA 1.40). Photobleaching was carried out with the built-in bleaching module by irradiating the region of interest (ROI) with 488-nm and 561-nm lasers at 100% laser power for 1 s. Time-lapse imaging was subsequently performed at 3-s intervals. Images were analyzed using ImageJ, and fluorescence intensities of each ROI were background-subtracted and normalized so that the prebleach value was set to 1 and the immediate postbleach value to 0. Fluorescence recovery was plotted over 30 s after bleaching to evaluate recovery kinetics.

### Statistical Analysis.

Microsoft Excel for Microsoft 365 MSO 2306 and GraphPad Prism v8.4.1 were used for data analysis and graph preparation. Data are presented as mean ± SD. Comparisons between two groups were performed using a two-tailed unpaired or paired Student’s *t* test, as appropriate. Comparisons among multiple groups were performed using one-way ANOVA followed by Dunnett’s multiple comparisons test. The statistical tests used for each analysis are indicated in the corresponding figure legends. A *P* value of < 0.05 was considered statistically significant.

## Supplementary Material

Appendix 01 (PDF)

Movie S1. **PM signals emerge at the bleb base during bleb expansion**. Time-lapse imaging of a bleb in Ba/F3 cells stained with CellMask, showing the appearance of PM–derived signals at the bleb base during bleb expansion (related to Fig. 1A). Scale bar, 2 μm.

Movie S2. **Three-dimensional CLEM reveals tubular SBIs at the bleb base**. Three-dimensional correlative light and electron microscopy (CLEM) analysis of a bleb in Ba/F3 cells, showing tubular PM invaginations continuous with the bleb base (related to Fig. 1D).

Movie S3. **FBP17 accumulates dynamically at the bleb base**. Time-lapse imaging of a bleb in HT1080 cells expressing GFP-FBP17, showing transient accumulation of FBP17 at the bleb base during bleb expansion (related to Fig. 2A). Scale bar, 2 μm.

Movie S4. **Rapid recruitment of FBP17 to nascent SBIs during bleb expansion**. High-speed time-lapse imaging of HT1080 cells expressing GFP-FBP17 and stained with CellMask, showing rapid recruitment of FBP17 to nascent SBIs during bleb expansion (related to Fig. 2C). Scale bar, 2 μm.

Movie S5. **Three-dimensional dynamics of FBP17-labeled SBIs**. Three-dimensional time-lapse imaging of a bleb in HT1080 cells expressing GFP-FBP17, showing the dynamic behavior of FBP17-labeled SBIs during bleb expansion (related to Fig. 2E). Scale bar, 5 μm.

Movie S6. **Cav1 accumulates at FBP17-labeled SBIs**. Time-lapse imaging of a bleb in HT1080 cells co-expressing GFP-Cav1 (green) and mCherry-FBP17 (magenta), showing Cav1 accumulation at FBP17-labeled SBIs (related to Fig. 3A). Scale bar, 2 μm.

Movie S7. **High-speed dynamics of Cav1 and FBP17 at SBIs**. High-speed time-lapse imaging of HT1080 cells co-expressing GFP-Cav1 and mCherry-FBP17, showing the dynamic recruitment of Cav1 and FBP17 to SBIs during bleb expansion (related to Fig. S4A). Scale bar, 2 μm.

Movie S8. **Cav1 is excluded from outward membrane protrusions**. Time-lapse imaging of M2 cells co-expressing GFP-Cav1 (green) and Scarlet-PD-L1 (magenta) before and after serum stimulation, showing that Cav1 is excluded from outward membrane protrusions such as filopodia and blebs (related to Fig. S4F). Scale bar, 5 μm.

Movie S9. **FRAP analysis reveals stable retention of Cav1 at SBIs**. FRAP analysis of SBIs in HT1080 cells co-expressing GFP-FBP17 (green) and mCherry-Cav1 (magenta). The bleached region is indicated by a red circle (related to Fig. 4A). Scale bar, 2 μm.

Movie S10. **Piezo1 accumulates at FBP17-labeled SBIs**. Time-lapse imaging of a bleb in HT1080 cells co-expressing GFP-FBP17 (green) and Piezo1-Halo (magenta), showing Piezo1 accumulation at FBP17-labeled SBIs (related to Fig. 4C). Scale bar, 2 μm.

Movie S11. **Piezo1 is excluded from outward membrane protrusions**. Time-lapse imaging of M2 cells co-expressing YFP-Piezo1 (green) and Scarlet-PD-L1 (magenta) before and after serum stimulation, showing that Piezo1 is excluded from outward membrane protrusions such as filopodia and blebs (related to Fig. S4J). Scale bar, 5 μm.

Movie S12. **Yoda1 increases Piezo1 localization at PM of expanding blebs**. Time-lapse imaging of YFP-Piezo1 dynamics at blebs before and after Yoda1 treatment, displayed as inverted projections, showing increased Piezo1 localization at the PM of expanding blebs after treatment (related to Fig. 4E). Scale bar, 5 μm.

## Data Availability

All study data are included in the article and/or supporting information.
