# Supplementary material for: Bleb expansion requires transient membrane invaginations that sequester curvature-preferring proteins
Source: Proc Natl Acad Sci U S A. 2026 May 20;123(21):e2534871123. doi: 10.1073/pnas.2534871123 (PMC13213987; doi:10.1073/pnas.2534871123)
Supplement: Supplementary file 1 — Appendix 01 (PDF) [file pnas.2534871123.sapp.pdf]

PNAS

Supporting Information for

Bleb expansion requires transient membrane invaginations that sequester curvature-preferring proteins

Yuki Maekawa, Saori R. Yoshii, Noboru Mizushima and Junichi Ikenouchi

Junichi Ikenouchi
Email: ikenouchi.junichi.033@m.kyushu-u.ac.jp

This PDF file includes:

Figures S1 to S5
Legends for Movies S1 to S12

Other supporting materials for this manuscript include the following:

Movies S1 to S12

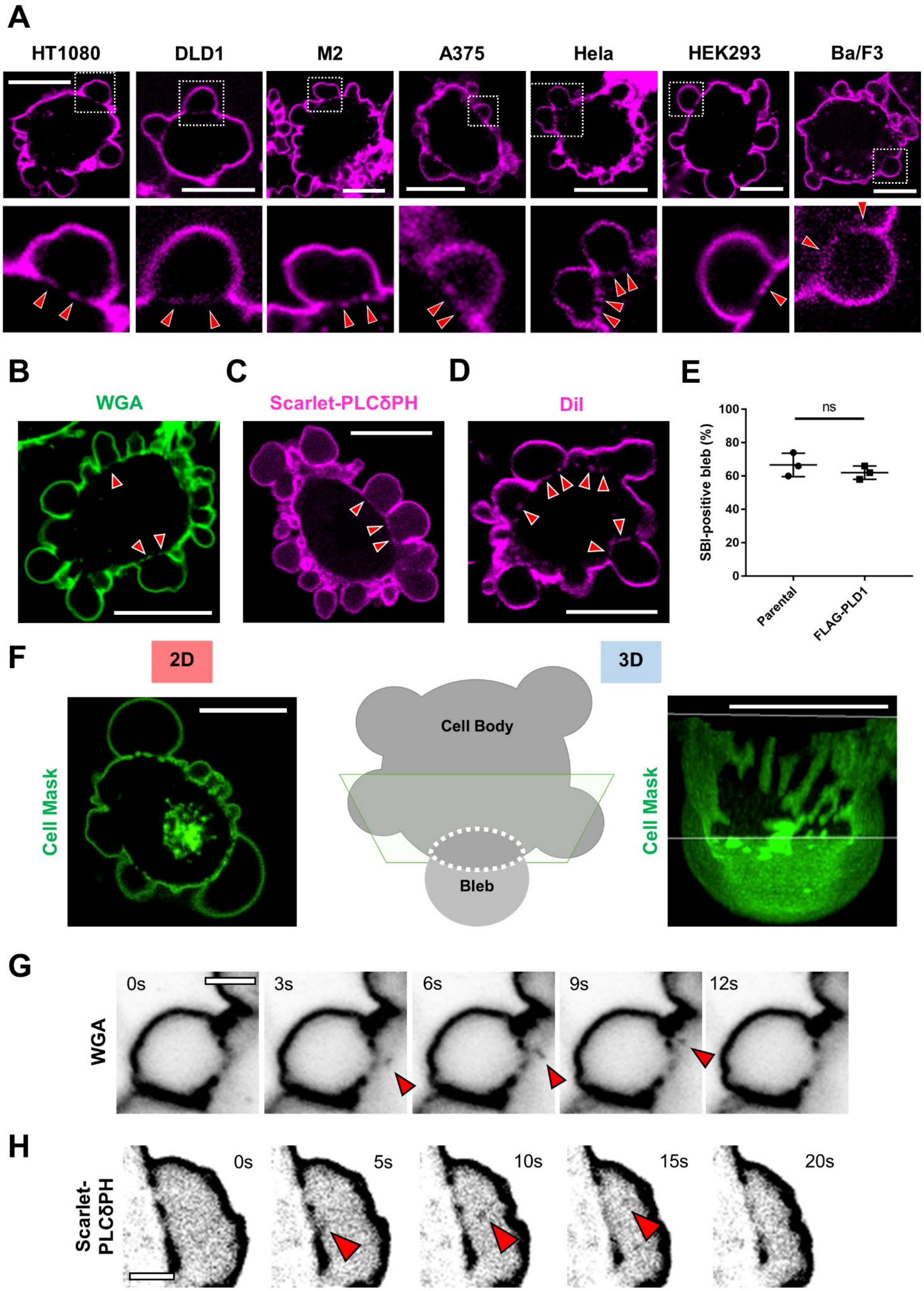
A
HT1080
DLD1
M2
A375
Hela
HEK293
Ba/F3
B
WGA
C
Scarlet-PLCδPH
D
DiI
E
SBI-positive bleb (%)
ns
Parental
FLAG-PLD1
F
2D
3D
Cell Mask
Cell Body
Bleb
Cell Mask
G
WGA
0s
3s
6s
9s
12s
H
Scarlet-PLCδPH
0s
5s
10s
15s
20s

Fig. S1. PM markers reveal tubular invaginations at the bleb base (related to Figure 1)

(A) Images of blebbing cells from multiple cell lines stained with CellMask. Insets show higher-magnification views of the boxed regions. Red arrowheads indicate PM signals at the bleb base. Blebs were analyzed in HT1080 (fibrosarcoma), DLD1 (colorectal cancer), M2 and A375 (melanoma), HeLa (cervical cancer), HEK293 (non-transformed kidney), and parental Ba/F3 cells (murine pro-B cells). Scale bar, 10 μm.
(B) Image of a blebbing HT1080 cell stained with FITC-WGA. Red arrowheads indicate PM signals at the bleb base. Scale bar, 10 μm.
(C) Image of a blebbing HT1080 cell expressing Scarlet-PLCδPH. Red arrowheads indicate PM signals at the bleb base. Scale bar, 10 μm.
(D) Image of a blebbing HT1080 cell stained with DiI. Red arrowheads indicate PM signals at the bleb base. Scale bar, 10 μm.
(E) Quantification of the frequency of SBI-positive blebs in parental Ba/F3 cells and FLAG-PLD1–expressing Ba/F3 cells labeled with CellMask. Blebs showing SBI-like PM invagination signals were expressed as a percentage of the total number of blebs examined. Fifty blebs were analyzed per experiment. Data are shown as mean ± SD (n = 3 independent experiments). Statistical significance was assessed using a two-tailed unpaired Student's t test.
(F) Single focal-plane (left) and three-dimensional reconstructed (right) images of a CellMask-stained HT1080 cell showing a bleb protruding toward the viewer. Tubular PM invaginations extend inward from the bleb base. Scale bar, 10 μm.
(G) Time-lapse image of a bleb in HT1080 cells stained with FITC-WGA. Red arrowheads indicate fragmented SBIs. Scale bar, 2 μm.
(H) Time-lapse images of a bleb in HT1080 cells expressing Scarlet-PLCδPH. Red arrowheads indicate fragmented SBIs. Scale bar, 2 μm.

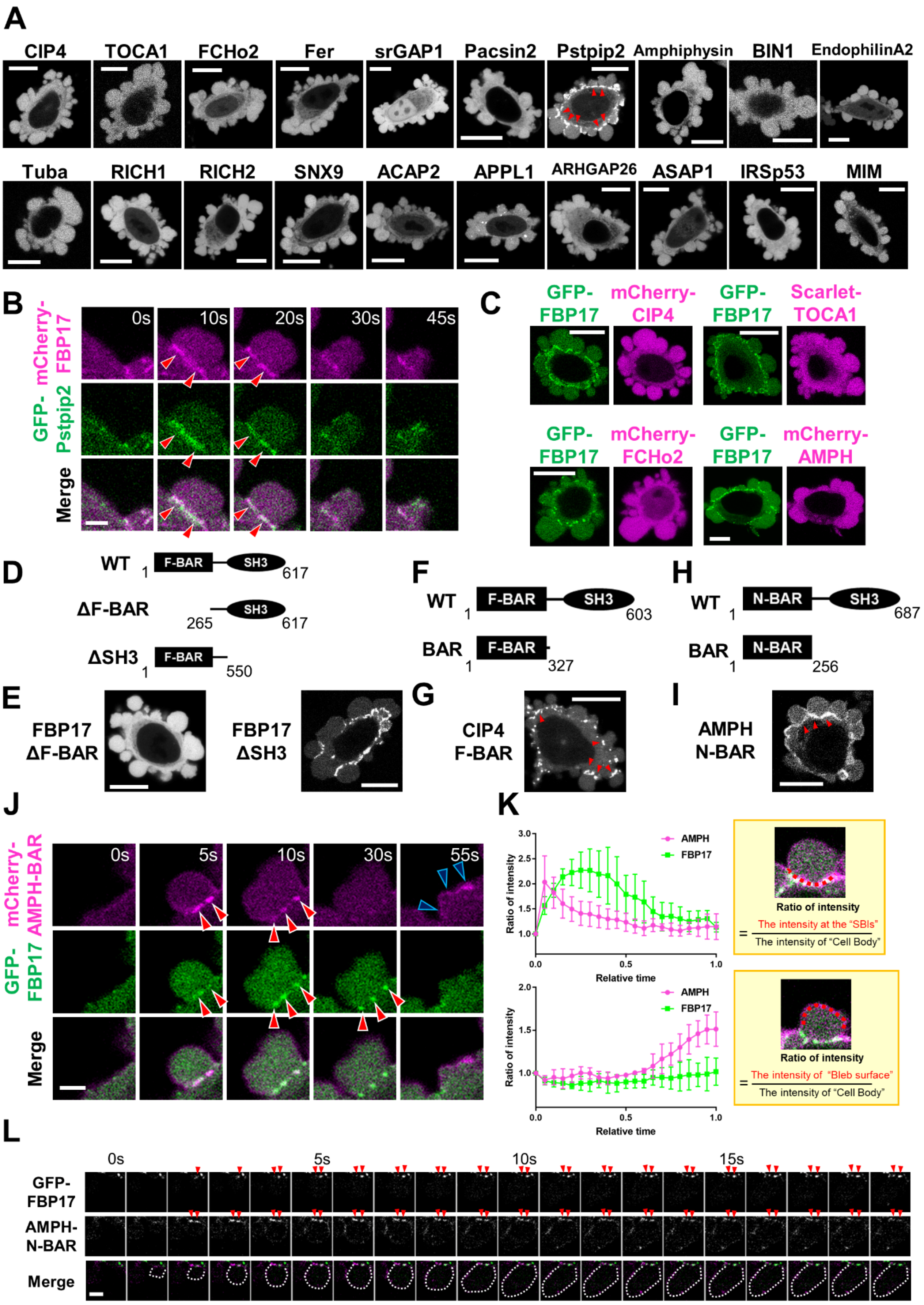
A
CIP4
TOCA1
FCHo2
Fer
srGAP1
Pacsin2
Pstpip2
Amphiphysin
BIN1
EndophilinA2
Tuba
RICH1
RICH2
SNX9
ACAP2
APPL1
ARHGAP26
ASAP1
IRSp53
MIM
B
mCherry-FBP17
GFP-Pstpip2
Merge
0s
10s
20s
30s
45s
C
GFP-FBP17
mCherry-CIP4
GFP-FBP17
Scarlet-TOCA1
GFP-FBP17
mCherry-FCHo2
GFP-FBP17
mCherry-AMPH
D
WT
F-BAR
SH3
1
617
ΔF-BAR
265
ΔSH3
550
F
603
BAR
327
H
N-BAR
687
256
E
FBP17 ΔF-BAR
FBP17 ΔSH3
G
CIP4 F-BAR
I
AMPH N-BAR
J
mCherry-AMPH-BAR
GFP-FBP17
0s
5s
10s
30s
55s
K
AMPH
FBP17
Ratio of intensity
Relative time
Ratio of intensity
= The intensity at the "SBIs" / The intensity of "Cell Body"
Ratio of intensity
= The intensity of "Bleb surface" / The intensity of "Cell Body"
L
0s
5s
10s
15s
GFP-FBP17
AMPH-N-BAR
Merge

Fig. S2. Localization of BAR-domain proteins in blebbing cells

(A) Representative images of HT1080 cells forming blebs transiently expressing fluorescently tagged BAR-domain proteins. The red arrowheads indicate regions of accumulation of the labeled proteins at SBIs formed at the base of the bleb. Scale bar, 10 μm.
(B) Time-lapse images of a bleb in HT1080 cells co-expressing GFP-Pstpip2 and mCherry-FBP17. Red arrowheads indicate the colocalization of FBP17 and Pstpip2 at the SBIs. Scale bar, 2 μm.
(C) Representative images of blebbing HT1080 cells transiently co-expressing GFP-FBP17 with mCherry-CIP4, FCHo2, AMPH, or Scarlet-TOCA1. Scale bar, 10 μm.
(D) Schematic diagram of FBP17 truncation constructs.
(E) Localization of GFP-FBP17 truncation constructs in HT1080 cells. Scale bar, 10 μm.
(F) Schematic diagram of CIP4 truncation constructs.
(G) Representative images of HT1080 cells expressing GFP-CIP4 F-BAR. Red arrowheads indicate CIP4 F-BAR enrichment at the SBIs. Scale bar, 10 μm.
(H) Schematic diagram of AMPH truncation constructs.
(I) Representative images of HT1080 cells expressing mCherry-AMPH-N-BAR. Red arrowheads indicate AMPH-N-BAR enrichment at the SBIs. Scale bar, 10 μm.
(J) Time-lapse images of a bleb in HT1080 cells co-expressing GFP-FBP17 and mCherry-AMPH-N-BAR. Red arrowheads indicate FBP17 or AMPH-N-BAR enrichment at the SBIs, whereas blue arrowheads mark AMPH-N-BAR accumulation at the bleb surface during retraction. Scale bar, 2 μm.
(K) Time-course analysis of molecular accumulation at the bleb surface and in SBIs. Fluorescence intensity ratios of GFP-FBP17 and mCherry-AMPH-N-BAR at the bleb surface or in SBIs were measured and normalized to the cell body. Data represent five independent blebs, and the mean ± SD are plotted for each time point.
(L) High-speed time-lapse imaging of an expanding bleb in HT1080 cells co-expressing GFP-FBP17 and mCherry-AMPH-N-BAR. Red arrowheads indicate FBP17 or AMPH-N-BAR enrichment at the SBIs. Scale bar, 2 μm.

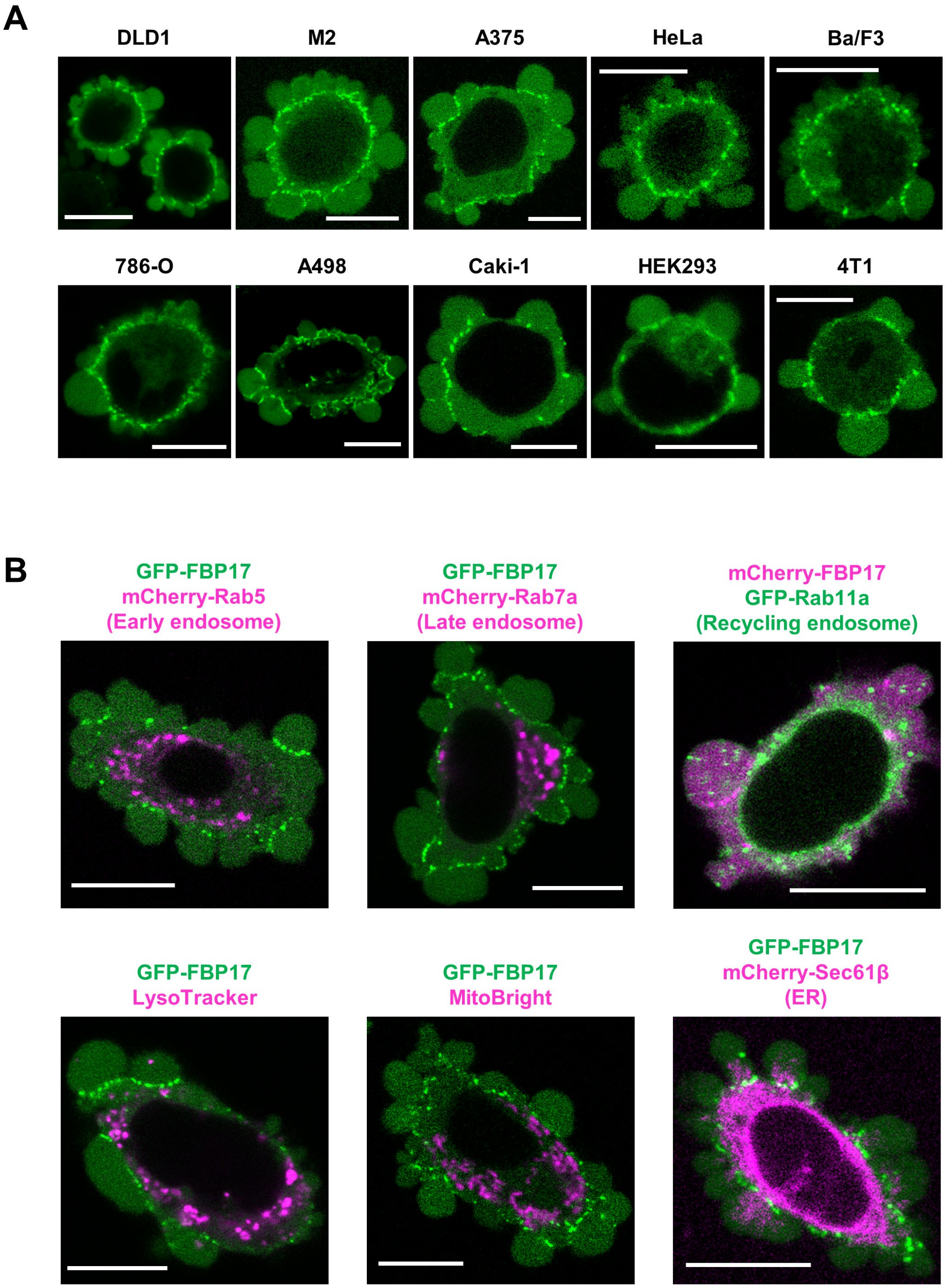

Fig. S3. Validation of FBP17 as an SBI marker (related to Figure 2)

(A) GFP-FBP17 localization in different cell lines. Blebs were analyzed in DLD1 cells (colorectal cancer), M2 and A375 cells (melanoma), HeLa cells (cervical cancer), Ba/F3 cells (murine pro-B cells), 786-O, A498 and Caki-1 cells (renal cell carcinoma), HEK293 cells (non-tumor kidney), and 4T1 cells (murine breast cancer). Scale bar, 10 µm.

(B) Comparison of FBP17-positive structures with various organelle markers in HT1080 cells. Cells expressing GFP- or mCherry-FBP17 were co-expressed with fluorescent organelle markers (Rab5: early endosome; Rab7a: late endosome; Rab11a: recycling endosome; Sec61β: endoplasmic reticulum) or stained with organelle dyes (LysoTracker: lysosome; MitoBright: mitochondria). Scale bar, 10 µm.

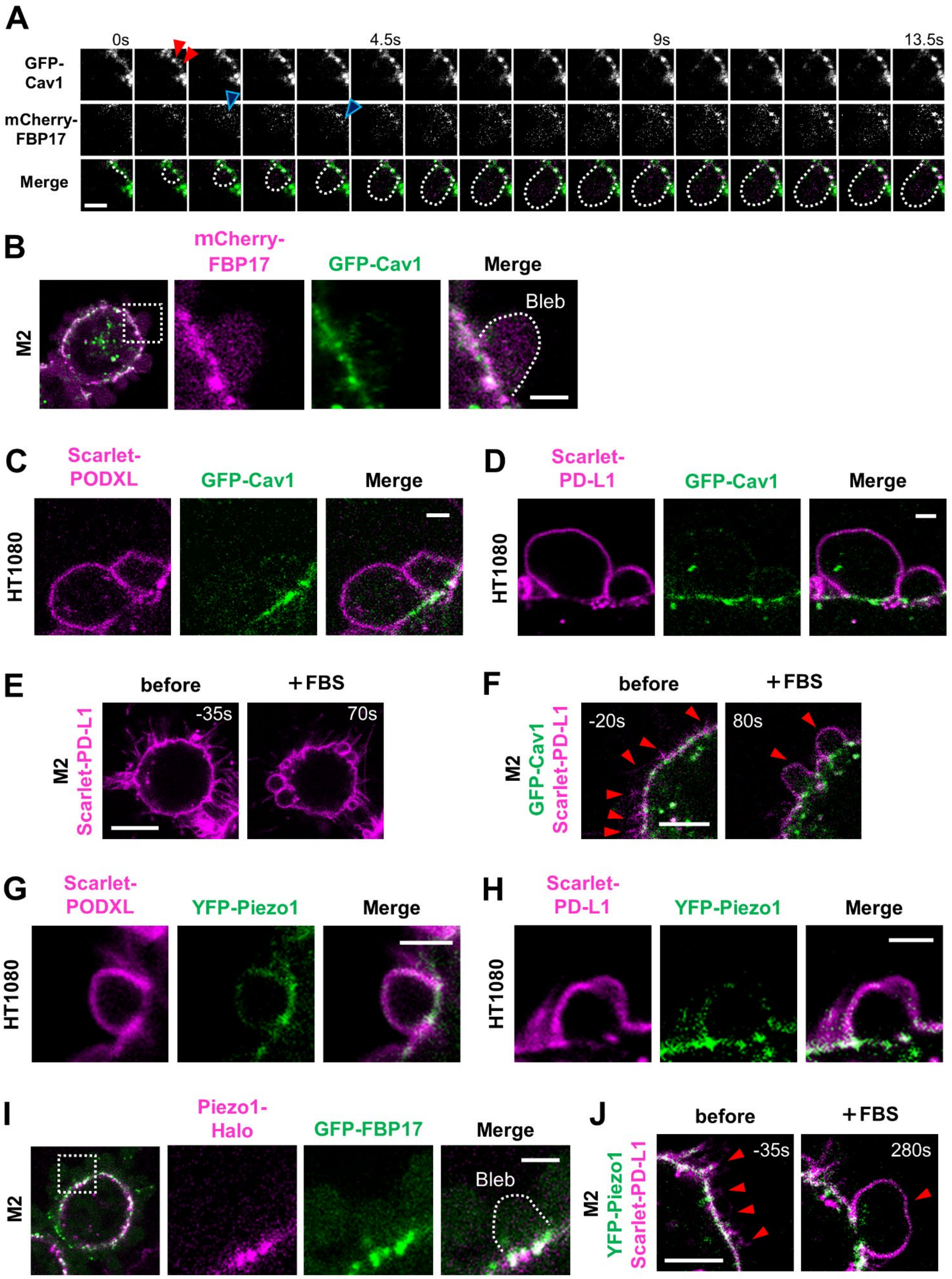

A
0s
4.5s
9s
13.5s
GFP-Cav1
mCherry-FBP17
Merge
B
mCherry-FBP17
GFP-Cav1
Merge
M2
Bleb
C
Scarlet-PODXL
GFP-Cav1
Merge
HT1080
D
Scarlet-PD-L1
GFP-Cav1
Merge
HT1080
E
before
+FBS
M2
Scarlet-PD-L1
-35s
70s
F
before
+FBS
M2
GFP-Cav1
Scarlet-PD-L1
-20s
80s
G
Scarlet-PODXL
YFP-Piezo1
Merge
HT1080
H
Scarlet-PD-L1
YFP-Piezo1
Merge
HT1080
I
Piezo1-Halo
GFP-FBP17
Merge
M2
Bleb
J
before
+FBS
M2
YFP-Piezo1
Scarlet-PD-L1
-35s
280s

Fig. S4. Cav1 and Piezo1 accumulate at the FBP17-labeled SBIs and are depleted from expanding blebs (related to Figures 3 and 4)

(A) High-speed time-lapse imaging of an expanding bleb in HT1080 cells co-expressing GFP-Cav1 and mCherry-FBP17. Red arrowheads mark Cav1 enrichment at the SBIs, whereas blue arrowheads mark FBP17 enrichment at the SBIs. Scale bar, 2 μm.
(B) Images of blebbing M2 cells co-expressing GFP-Cav1 and mCherry-FBP17 with a magnified view of a single bleb (white dotted line). Scale bar, 2 μm.
(C) Images of a bleb in HT1080 cells co-expressing GFP-Cav1 and Scarlet-PODXL. Scale bar, 2 μm.
(D) Images of a bleb in HT1080 cells co-expressing GFP-Cav1 and Scarlet-PD-L1. Scale bar, 2 μm.
(E) M2 cells expressing Scarlet-PD-L1 stimulated with serum (see Materials and Methods) to induce bleb formation. Serum was added at t = 0 s. Scale bar, 10 μm.
(F) Localization of Cav1 and PD-L1 before and after serum stimulation in M2 cells. Red arrowheads indicate that Cav1 is excluded from PM protrusions such as filopodia and blebs. Scale bar, 5 μm.
(G) Images of a bleb in HT1080 cells co-expressing YFP-Piezo1 and Scarlet-PODXL. Scale bar, 2 μm.
(H) Images of a bleb in HT1080 cells co-expressing YFP-Piezo1 and Scarlet-PD-L1. Scale bar, 2 μm.
(I) Images of blebbing M2 cells co-expressing GFP-FBP17 and Piezo1-Halo with a magnified view of a single bleb (white dotted line). Scale bar, 2 μm.
(J) Localization of Piezo1 and PD-L1 before and after serum stimulation in M2 cells. Red arrowheads indicate that Piezo1 is excluded from PM protrusions such as filopodia and blebs. Scale bar, 5 μm.

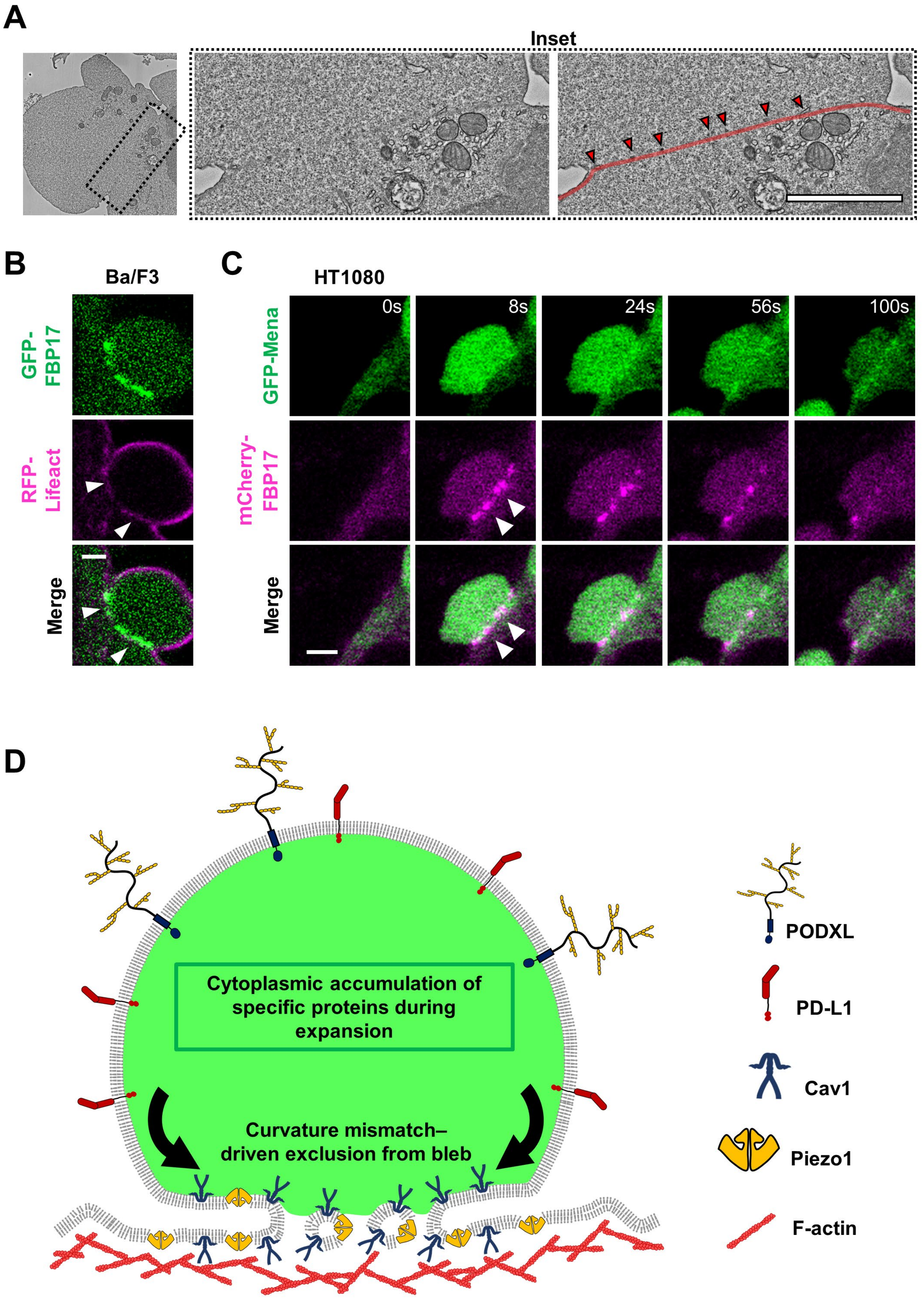
A
Inset
B
Ba/F3
GFP-FBP17
RFP-Lifeact
Merge
C
HT1080
0s
8s
24s
56s
100s
GFP-Mena
mCherry-FBP17
Merge
D
Cytoplasmic accumulation of specific proteins during expansion
Curvature mismatch–driven exclusion from bleb
PODXL
PD-L1
Cav1
Piezo1
F-actin

Fig. S5. Mechanism of SBI formation driven by curvature-preferring proteins at the bleb base and cytoplasmic proteins enriched in the bleb compartment

(A) CLEM images of a bleb in Ba/F3 cells stably expressing phospholipase D1 (reproduced from Fig. 1C). Insets show higher-magnification views of the boxed region. Red arrowheads mark SBIs, and red shading highlights the residual actin cortex remaining at the original cell boundary after membrane detachment. Scale bars, 2 μm.
(B) Images of a bleb in Ba/F3 cells co-expressing GFP-FBP17 and RFP-Lifeact. White arrowheads indicate FBP17-labeled SBIs adjacent to the Lifeact-positive residual actin cortex. Scale bar, 2 μm.
(C) Time-lapse images of a bleb in HT1080 cells co-expressing mCherry-FBP17 and GFP-Mena. White arrowheads indicate FBP17-labeled SBI localized at the interface with the Mena-enriched region inside the bleb. Scale bar, 2 μm.
(D) Schematic illustration summarizing the composition and formation mechanism of the SBIs. Synergistic action between inward pulling of the membrane by cytoplasmic protein enrichment inside the bleb and accumulation of curvature-preferring proteins at the bleb base may drive the formation of SBIs.

Movie S1 (separate file). PM signals emerge at the bleb base during bleb expansion

Time-lapse imaging of a bleb in Ba/F3 cells stained with CellMask, showing the appearance of PM–derived signals at the bleb base during bleb expansion (related to Fig. 1A). Scale bar, 2 μm.

Movie S2 (separate file). Three-dimensional CLEM reveals tubular SBIs at the bleb base

Three-dimensional correlative light and electron microscopy (CLEM) analysis of a bleb in Ba/F3 cells, showing tubular PM invaginations continuous with the bleb base (related to Fig. 1D).

Movie S3 (separate file). FBP17 accumulates dynamically at the bleb base

Time-lapse imaging of a bleb in HT1080 cells expressing GFP-FBP17, showing transient accumulation of FBP17 at the bleb base during bleb expansion (related to Fig. 2A). Scale bar, 2 μm.

Movie S4 (separate file). Rapid recruitment of FBP17 to nascent SBIs during bleb expansion

High-speed time-lapse imaging of HT1080 cells expressing GFP-FBP17 and stained with CellMask, showing rapid recruitment of FBP17 to nascent SBIs during bleb expansion (related to Fig. 2C). Scale bar, 2 μm.

Movie S5 (separate file). Three-dimensional dynamics of FBP17-labeled SBIs

Three-dimensional time-lapse imaging of a bleb in HT1080 cells expressing GFP-FBP17, showing the dynamic behavior of FBP17-labeled SBIs during bleb expansion (related to Fig. 2E). Scale bar, 5 μm.

Movie S6 (separate file). Cav1 accumulates at FBP17-labeled SBIs

Time-lapse imaging of a bleb in HT1080 cells co-expressing GFP-Cav1 (green) and mCherry-FBP17 (magenta), showing Cav1 accumulation at FBP17-labeled SBIs (related to Fig. 3A). Scale bar, 2 μm.

Movie S7 (separate file). High-speed dynamics of Cav1 and FBP17 at SBIs

High-speed time-lapse imaging of HT1080 cells co-expressing GFP-Cav1 and mCherry-FBP17, showing the dynamic recruitment of Cav1 and FBP17 to SBIs during bleb expansion (related to Fig. S4A). Scale bar, 2 μm.

Movie S8 (separate file). Cav1 is excluded from outward membrane protrusions

Time-lapse imaging of M2 cells co-expressing GFP-Cav1 (green) and Scarlet-PD-L1 (magenta) before and after serum stimulation, showing that Cav1 is excluded from outward membrane protrusions such as filopodia and blebs (related to Fig. S4F). Scale bar, 5 μm.

Movie S9 (separate file). FRAP analysis reveals stable retention of Cav1 at SBIs

FRAP analysis of SBIs in HT1080 cells co-expressing GFP-FBP17 (green) and mCherry-Cav1 (magenta). The bleached region is indicated by a red circle (related to Fig. 4A). Scale bar, 2 μm.

Movie S10 (separate file). Piezo1 accumulates at FBP17-labeled SBIs

Time-lapse imaging of a bleb in HT1080 cells co-expressing GFP-FBP17 (green) and Piezo1-Halo (magenta), showing Piezo1 accumulation at FBP17-labeled SBIs (related to Fig. 4C). Scale bar, 2 μm.

Movie S11 (separate file). Piezo1 is excluded from outward membrane protrusions

Time-lapse imaging of M2 cells co-expressing YFP-Piezo1 (green) and Scarlet-PD-L1 (magenta) before and after serum stimulation, showing that Piezo1 is excluded from outward membrane protrusions such as filopodia and blebs (related to Fig. S4J). Scale bar, 5 μm.

Movie S12 (separate file). Yoda1 increases Piezo1 localization at PM of expanding blebs

Time-lapse imaging of YFP-Piezo1 dynamics at blebs before and after Yoda1 treatment, displayed as inverted projections, showing increased Piezo1 localization at the PM of expanding blebs after treatment (related to Fig. 4E). Scale bar, 5 μm.
